# Impact of fig mosaic virus on the life history traits and fitness trade-offs of its eriophyid mite vector, *Aceria ficus*

**DOI:** 10.1038/s41598-026-46471-4

**Published:** 2026-04-06

**Authors:** Negar Daneshnia, Keramatollah Izadpanah, Yaghoub Fathipour, Mohammad Ali Akrami, Mohammad Mehdi Faghihi, Mohammad Mehrabadi

**Affiliations:** 1 Department of Entomology, Faculty of Agriculture, Tarbiat Modares University, P.O. Box 14115-336, Tehran, Iran; 2 Plant Virology Research Centre, College of Agriculture, Shiraz University, Shiraz, Iran; 3 Department of Plant Protection, College of Agriculture, Shiraz University, Shiraz, Iran; 4 Department of Plant Protection Research, Fars Agricultural and Natural Resources Research and Education Center, AREEO, Zarghan, Iran

**Keywords:** Vector biology, Fig mite, Vector-plant virus interaction, Life history traits, Entomology, Zoology

## Abstract

Most plant viruses depend upon arthropod vectors for their transmission among plant hosts. There are shreds of evidence of insect vectors and host manipulations by plant viruses for facilitating virus transmission. Fig mosaic disease is a globally distributed viral plant disease for which only fig mosaic virus (FMV) has been identified as the causal agent. In this study, we investigated the effect of FMV on the fitness of its mite vector using an age-stage two-sex life table approach, which provided detailed life stage durations of each nymph and adult (both sexes). To do this, the life history traits of the FMV-viruliferous (V) and non-viruliferous (NV) mite colonies on the healthy fig leaves and FMV-viruliferous mites on FMV-infected fig leaves (VL) were analyzed. Seventy pairs of adult mites from each colony (same age) were released on uninfected and infected fig leaves in a Petri dish, and placed in a growth chamber at 28 ± 2 °C and 12:12 (L: D) photoperiod. Our results showed that the non-viruliferous mites survived longer, produced higher number of offspring, and showed less mortality, while the viruliferous mites completed their pre-adult development faster than the non-viruliferous mites. Despite these differences, there were no significant differences in the population growth parameters of the viruliferous mites compared to the non-viruliferous mites. This suggests that FMV may exert subtle fitness trade-offs on its vector, balancing potential fitness costs to ensure successful transmission.

**Supplementary Information:**

The online version contains supplementary material available at 10.1038/s41598-026-46471-4.

## Introduction

Vector-borne plant pathogens, including viruses and bacteria, rely on vectors for transmission between susceptible hosts. These pathogens frequently cause economically significant and devastating plant diseases, with their transmission and spread heavily dependent on the abundance, fitness, and behavior of their vectors^1–3^. To successfully propagate, vector-borne pathogens are frequently under selection to modify or manipulate their vectors, directly or indirectly, in ways that enhance transmission and dispersal^4–7^. Many of these manipulations are mediated indirectly through pathogen-induced changes to the host plant’s phenotype, making infected plants more attractive or palatable to vectors, thus enhancing their feeding and association with the infected host^8–10^. However, evidence also shows that plant pathogens can directly influence vector performance and behavior, increasing transmission probabilities^11,12^. These direct and indirect effects may simultaneously occur to shape vector-pathogen dynamics over evolutionary time^13^. For instance, plant viruses can induce physiological and biochemical changes in infected plants, making them more attractive or palatable to vectors, thereby increasing feeding and transmission rates^8,14^. Additionally, some viruses directly influence the fitness, development, or behavior of their vectors, creating intricate interactions that range from mutualistic to parasitic^16–19^. While mutualistic interactions can enhance vector fitness and benefit the virus and its vector, parasitic interactions may impose fitness costs on the vector, raising questions about the evolutionary trade-offs between vector fitness and effective virus transmission^19^.

Arthropods, including insects and mites, are among the most critical vectors of plant pathogens^1^. While insect vectors have been extensively studied, there is less focus on the role of eriophyid mites, which are important vectors for virus transmission in plants. Eriophyid mites (Eriophyoidea) are entirely phytophagous, and several species are recognized as significant economic pests. These mites have a specialized piercing feeding mode, using their stylets to penetrate plant cells and feed on cellular contents, making them effective vectors of various plant viruses^20^. Specific relationships often exist between eriophyid mites and the viruses they transmit. A well-studied example is the association between eriophyid mites like *Aceria ficus* and Fig mosaic virus (FMV), the causal agent of fig mosaic disease (FMD)^21,22^.

FMD is a major global disease affecting fig (Ficus carica) crops, causing significant economic losses in fig production worldwide^23^. Infected trees often exhibit mosaic-like symptoms on leaves and fruits, coupled with defoliation, fruit deformity, and premature fruit drop^24^. FMV is a segmented, negative-sense, single-stranded RNA virus classified under the genus Emaravirus of the order Bunyavirales^25^. The virus is transmitted predominantly by the eriophyid mite *A. ficus*, with a reported transmission rate of approximately 70% ^26^. In addition to vector-mediated transmission, FMV spreads through vegetative fig propagation^25^.

While plant virus-mediated effects on insect vectors and plant hosts have been widely studied, such interactions remain poorly understood in the context of mite vectors and their associated viruses. Research has documented the capacity of plant viruses to manipulate insect vector fitness, behavior, and population dynamics to ensure efficient transmission^3,9^. However, there is no clear evidence of similar manipulative effects on eriophyid mites. As eriophyid mites play a key role in the epidemiology of certain plant viruses, understanding their fitness and population dynamics when viruliferous is essential to elucidating mite-virus interactions.

This study investigates whether FMV infection influences the fitness and life-history traits of its eriophyid mite vector, *A. ficus*. We used a comprehensive age-stage two-sex life table approach to assess critical parameters of mite performance, including survival, fecundity, and development in FMV-viruliferous and non-viruliferous mite populations. Our findings reveal that FMV infection affects some fitness traits; however, these effects are not substantial enough to influence key population growth parameters.

## Materials and methods

### Mite collection and rearing

The eriophyid fig mites (FM) were collected from healthy (virus-free) fig trees in a fig orchard (Green cultivar, Shiraz, Fars province, Iran) during the summer of 2022–2023. Microscopic slides were prepared, and the mites were identified based on their morphological characteristics and genitalia, the main characteristic for distinguishing females and males. It was also found that the size of the females is larger than that of the males, and this characteristic was used for faster identification of males and females, next confirmed by the adult biology. The collected FM were identified and sexed with morphological characteristics and reared for five generations on healthy fig seedlings (Green cultivar, purchased from Shiraz University plant nursery, Shiraz, Iran) within metal-frame cages covered by insect-proof net in a controlled greenhouse environment (25 ± 4 °C, 60 ± 5% RH, and a photoperiod of 14:10 h), in Shiraz University. After one month, fig seedlings and mites were confirmed to be non-viruliferous using the PCR method described below. The non-viruliferous mites (NV) were then used to generate colonies. The mite and host plants were occasionally screened during the study to ensure they remained virus-free.

### Generation of viruliferous and non-viruliferous mite populations

To develop non-viruliferous (NV) and viruliferous (V) mite populations, non-viruliferous mites were pooled to establish a non-viruliferous colony and reared on healthy plants. A population of non-viruliferous mites was also transferred to FMV-infected plants (8-leaf growth stage for 12 h) to create a viruliferous colony, which was reared separately to prevent population mixing. One month later, the infection status of the mite colonies and host plants was assessed using RT-PCR. The infection status of the colonies was also regularly monitored throughout the study. The viruliferous mites were also reared on the healthy (VL) plants for two generations to compare the effect of healthy and virus-infected plants on the mites.

#### FMV detection in the mite vector and host plant

For virus infection status of the seedlings, total RNA was extracted from the leaves using RNA extraction kit (RNeasy Kit, Denazist). To determine if mites were harbored FMV, total RNA was extracted from 30 mites using the same kit. The extracted RNA quality was checked using a Nanodrop (ND-1000 Spectrophotometer). Then, cDNA was synthesized using the High-Capacity cDNA Reverse Transcriptase kit (Sinacolon First Strand cDNA Synthesis kit) with 2.0 µg RNA as template. PCR was performed using specific primers targeting FMV RNA1 (E5s: CGGTAGCAAATGGAATGAAA, and E5a: AACACTGTTTTTGCGATTGG)^27^. The PCR conditions were 95 °C (3 min), followed by 35 cycles of 14 °C (30 s), 53 °C (45 s), and final melting curve at 72 °C (1 min).

#### Fitness assays of viruliferous and non-viruliferous A. ficus

The performance of *A. ficus* mites was investigated using the two-sex life table method in the presence and absence of FMV. To do this, seventy pairs of the viruliferous FM adults (less than 12 h old) were placed on uninfected fig leaf discs or FMV-infected fig leaf discs surrounded with a thinned strip of cotton to inhibit mites escaping from Petri dishes (90 mm in diameter and 15 mm in height) and placed in a growth chamber at 28 ± 2 °C, photoperiod 12:12 h (L: D). Seventy pairs of the non-viruliferous FM adults were also placed on uninfected fig leaf discs and treated the same. After 24 h, the adult mites were collected from the leaf surface, and only one egg was left in each Petri dish. Developmental time and mortality rate were measured for each replicate until the adults emerged. At the next stage, a male mite was selected from the colony and placed in Petri dishes with a female mite, and then the daily fecundity and longevity were recorded. Premature duration stage, mortality, and total number of deposited eggs of *A. ficus* were counted daily to estimate life table parameters.

Raw data of individuals were analyzed according to the age-stage, two-sex life table theory^28^. The age-stage specific survival rate (*s*_*xj*_) (where *x* = age in days and _*j*_ = stage; ), the age-stage specific fecundity (*f*_*xj*_), the age-specific survival rate (*l*_*x*_), the age-specific fecundity (*m*_*x*_), and the population parameters (*r*, the intrinsic rate of increase; *λ*, the finite rate of increase, *λ* = *e*^*r*^; *R*_0_, the net reproductive rate; *T*, the mean generation time) were calculated. Because the life table study is highly time-consuming and replication is impractical, we used the bootstrap method^29^ to estimate the variances and standard errors of the life table parameters. The mean generation time (*T*) was defined as the length that a population needs to increase to *R*_0_ fold of its size at the stable age-stage distribution and was calculated as *T* = (ln *R*_0_)*/r*. The age-stage life expectancy (*e*_*xj*_) for individuals of age *x* and stage *j* was calculated according to the method described in ^28^. To ease the raw data analysis and then compile them, a computer program, TWOSEX-MS Chart for the age-stage, was used for two-sex life table analysis^30^. To compare non-viruliferous populations of the same stage, we used the paired bootstrap test (*p* < 0.05) at 5% significance level.

## Results

### FMV detection in A. ficus mites and its host fig plants

**Fig. 1:**
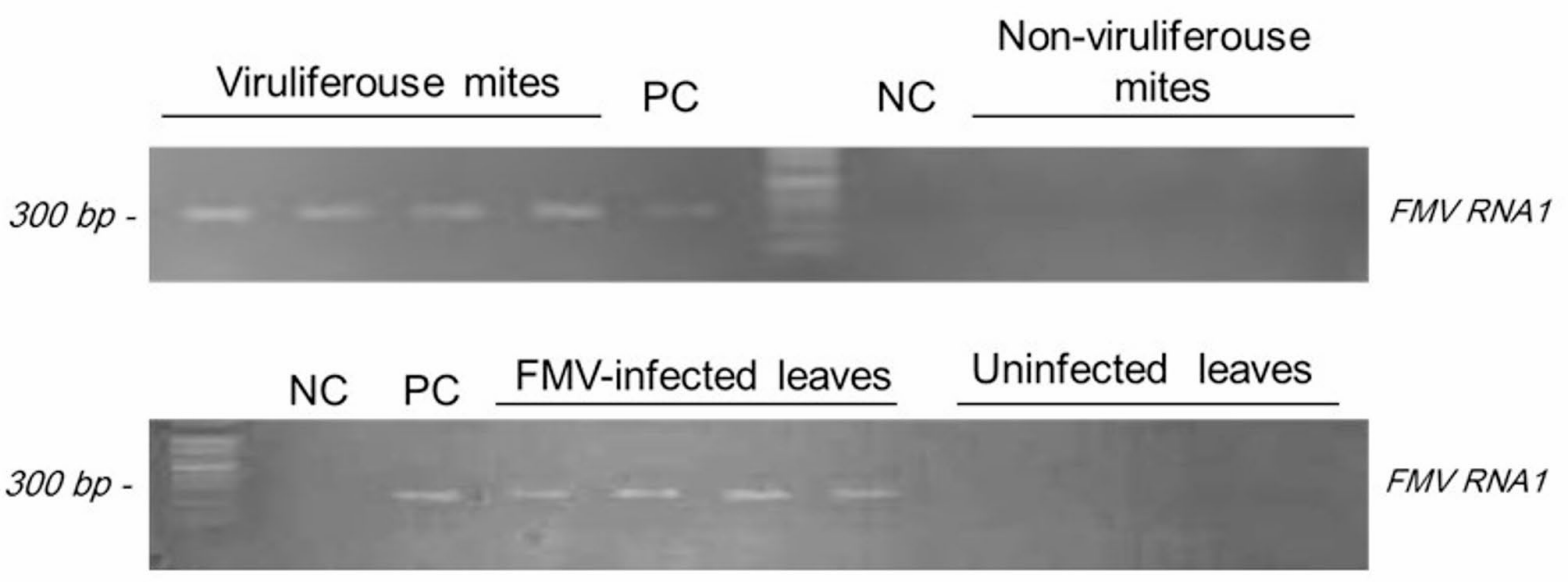
Detection of FMV in *Aceria ficus* individuals and fig leaves. RT-PCR analysis of RNA extracted from mites (**A**) fig leaves and specific primers targeting FMV RNA1.

To detect FMV infection, RNA samples were extracted from both mites and fig leaves. The RT-PCR test indicated that FMV was present in the viruliferous mites and infected leaves, while it was absent in the non-viruliferous mites and uninfected leaves (Fig. 1).

### Effect of FMV on the fitness of *A. ficus*

#### Duration of different developmental stages and survival of viruliferous and non-viruliferous *A. ficus*

**Fig. 2:**
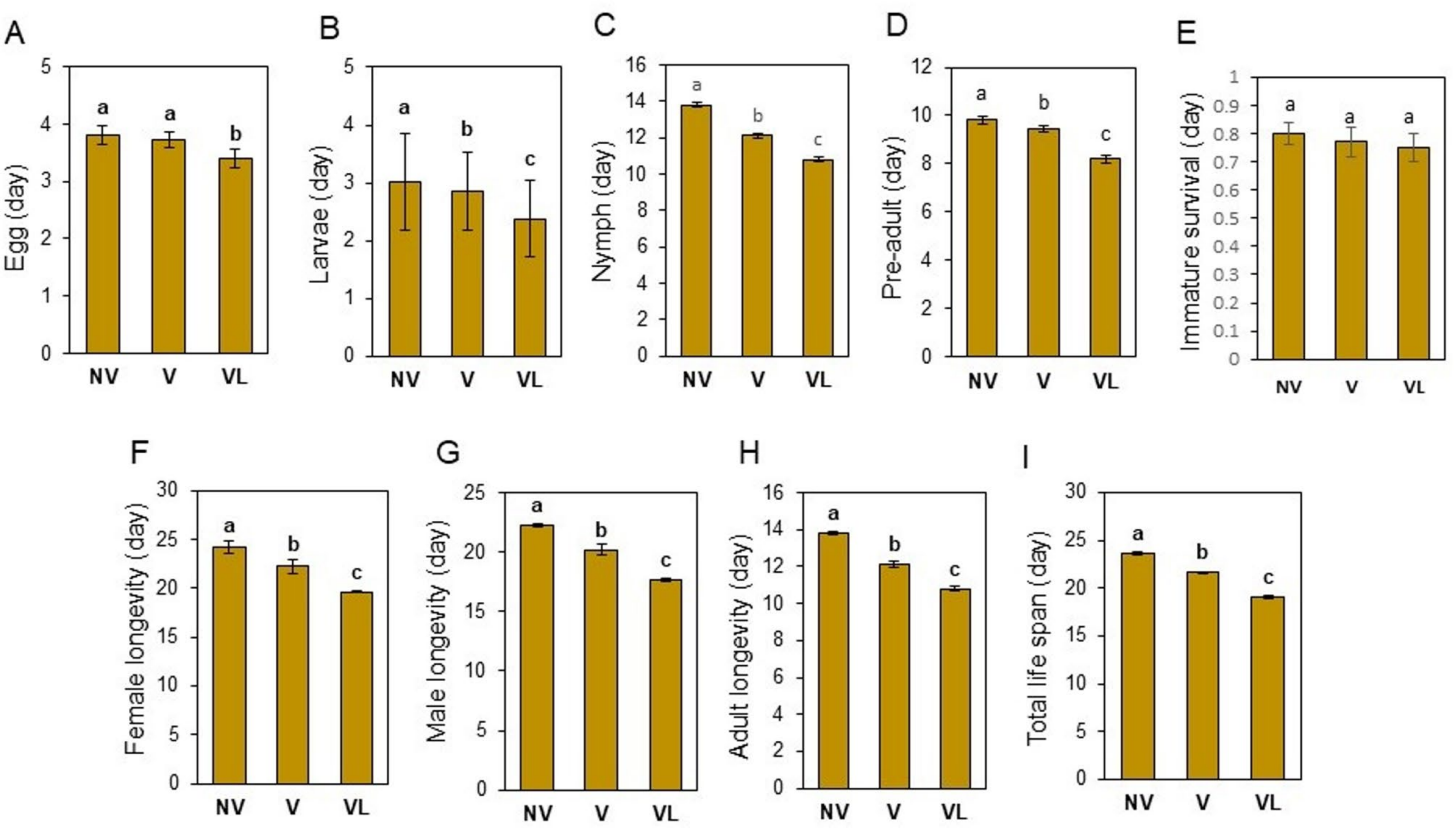
Developmental parameters (means ± SE) of non-viruliferous *Aceria ficus* reared on healthy fig leaves (NV), viruliferous mites reared on healthy fig leaves (V), and viruliferous mites reared on FMV-infected fig leaves (VL) calculated by using an age-stage, two-sex life table. Comparison of eggs (**A**), larvae (**B**), nymphs (**C**), pre-adult duration of progeny (**D**), immature survival (**E**), female longevity (**F**), male longevity (**G**), adult longevity (**H**), and total life span (**I**). Different letters indicate significantly different means, using a paired bootstrap test (*P* < 0.05).

The durations of different immature life stages in progeny from viruliferous and non-viruliferous mites are shown in Fig. 2A-D. The results show that the presence of FMV resulted in shorter (faster) development of immature stages (0.75 ± 0.05 day), while there were no differences in the survival rate of immature stages (*P* < 0.05) (Fig. 2E). The male and female longevity of adult mites was significantly different between viruliferous and non-viruliferous populations (*P* < 0.05) (Fig. 2F-H). The non-viruliferous adults reared on healthy fig leaves tended to have the greatest longevity (13.81 ± 0.12 days), followed by viruliferous mites fed on healthy fig leaves (12.12 ± 0.13 days) (*P* < 0.05), while the shortest longevity (10.81 ± 0.14 days) was observed in the viruliferous mites fed on FMV-infected fig leaves (Fig. 2F-H). Moreover, the total life span duration of *A. ficus* differed among different populations (Fig. 2I). The non-viruliferous mites showed the longest period (23.62 ± 0.13 days). The shortest period (19.1 ± 0.15 days) was observed in the viruliferous mites fed on FMV-infected fig leaves (*P* < 0.05) (Fig. 2I). The total life span of viruliferous mites fed on healthy fig leaves (21.6 ± 0.13 days) was longer than that of viruliferous mites fed on FMV-infected fig leaves (19.1 ± 0.15 days) (*P* < 0.05) (Fig. 2I).

### Reproduction parameters of viruliferous and non-viruliferous A. ficus

**Fig. 3:**
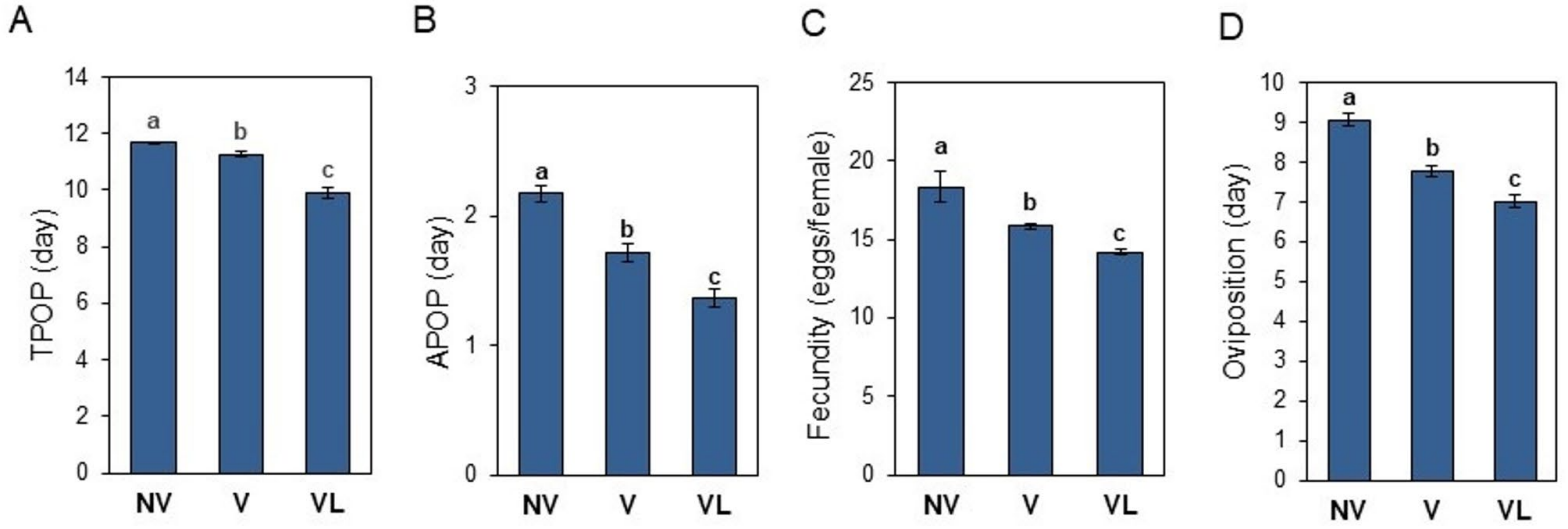
Reproduction parameters (means ± SE) of non-viruliferous (NV), viruliferous reared on healthy fig leaves (V), and viruliferous reared on FMV-infected fig leaves (VL) of *Aceria ficus* calculated by using an age-stage, two-sex life table. Comparison of the mean preoviposition period (TPOP) (**A**), adult preoviposition period (APOP) (**B**), fecundity (**C**), and oviposition rate (**D**). Different letters indicate significantly different means, using a paired bootstrap test (*P* < 0.05).

The longest preoviposition period (APOP) which is the period between the emergence of an adult female and her first oviposition, was observed in the progeny from the non-viruliferous mites (2.17 ± 0.06 days) followed by viruliferous mites feed on healthy fig leaves (1.72 ± 0.07 days) and viruliferous mites (1.36 ± 0.07 days) fed on FMV-infected fig leaves (*P* < 0.05) (Fig. 3A). The total preoviposition period (TPOP) which is the time interval from the birth of a female individual (from the egg stage) to the beginning of oviposition also showed similar trend in which the non-viruliferous mites had the longest period (11.66 ± 0.08 days), while the viruliferous mites feed on FMV-infected fig leaves had the shortest period (9.91 ± 0.13 days) (*P* < 0.05) (Fig. 3B). Fecundity (Fig. 3C) and oviposition days (Fig. 3D) also showed similar trend, where the non-viruliferous mites had the highest values [18.36 ± 0.09 (egg/female) and 9.07 ± 0.17 (day), respectively] (*P* < 0.05).

**Fig. 4:**
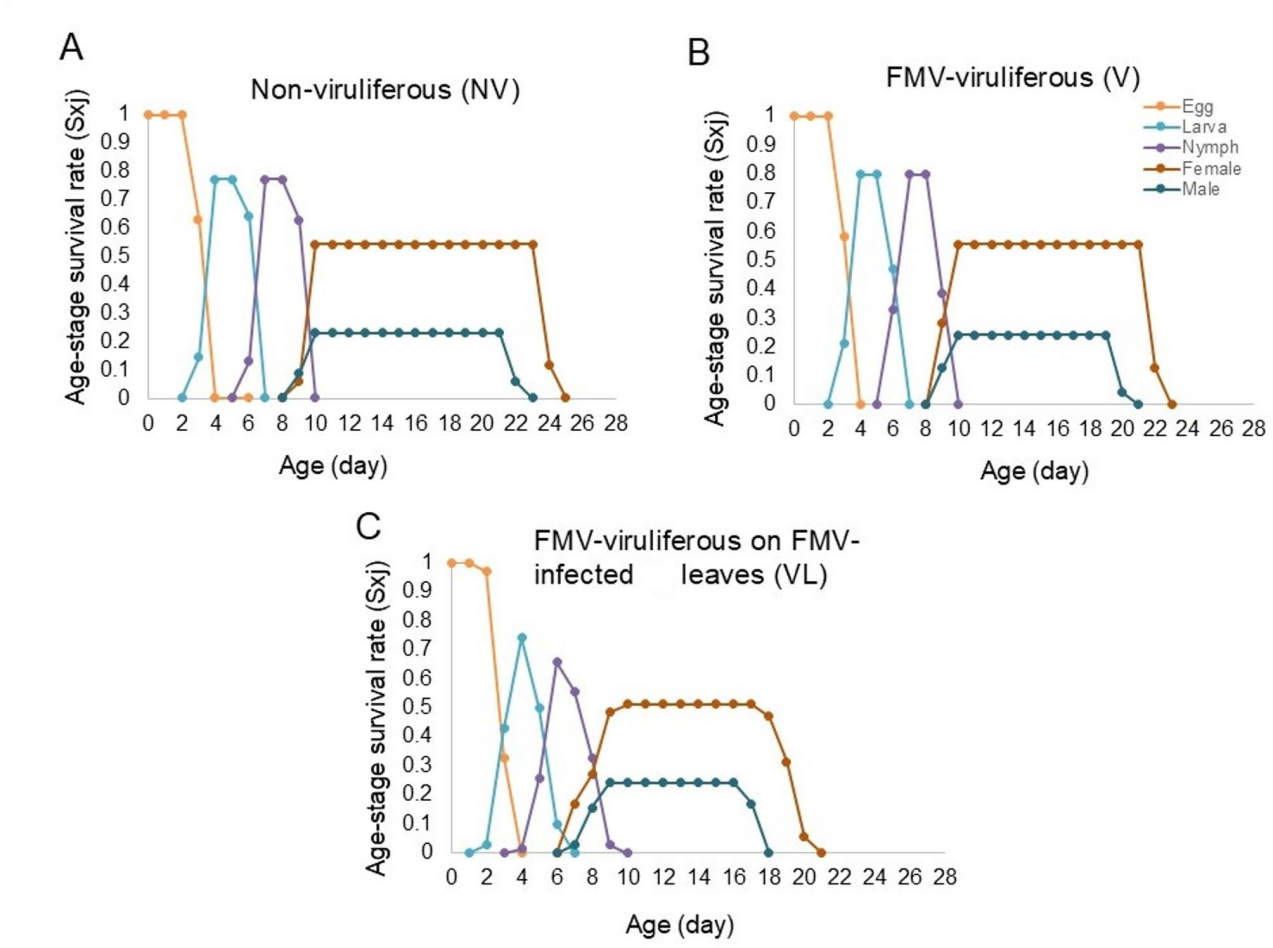
Effect of FMV on age-stage survival rate (Sxj) of *Aceria ficus*. (**A**) Non-viruliferous (NV), (**B**) viruliferous (V), and (**C**) viruliferous mite reared on FMV-infected fig leaves (VL).

The age-stage-specific survival rates (s_*xj*_) of *A. ficus* in different populations showed differences in survivorship and stage differentiation (Fig. 4A-C). The initiation and termination of each stage are shown in the survival curves and the overlap between different stages is attributable to the variable developmental rates among individuals (*P* < 0.05) (Fig. 4A-C). Adult progeny from the non-viruliferous mites survived longer than those from other populations. The shortest male (17.7 ± 0.11 days) and female survivals (19.63 ± 0.13 day) were observed in the adult progeny from the viruliferous mites fed on FMV-infected fig leaves (*P* < 0.05) (Fig. 4C).

**Fig. 5:**
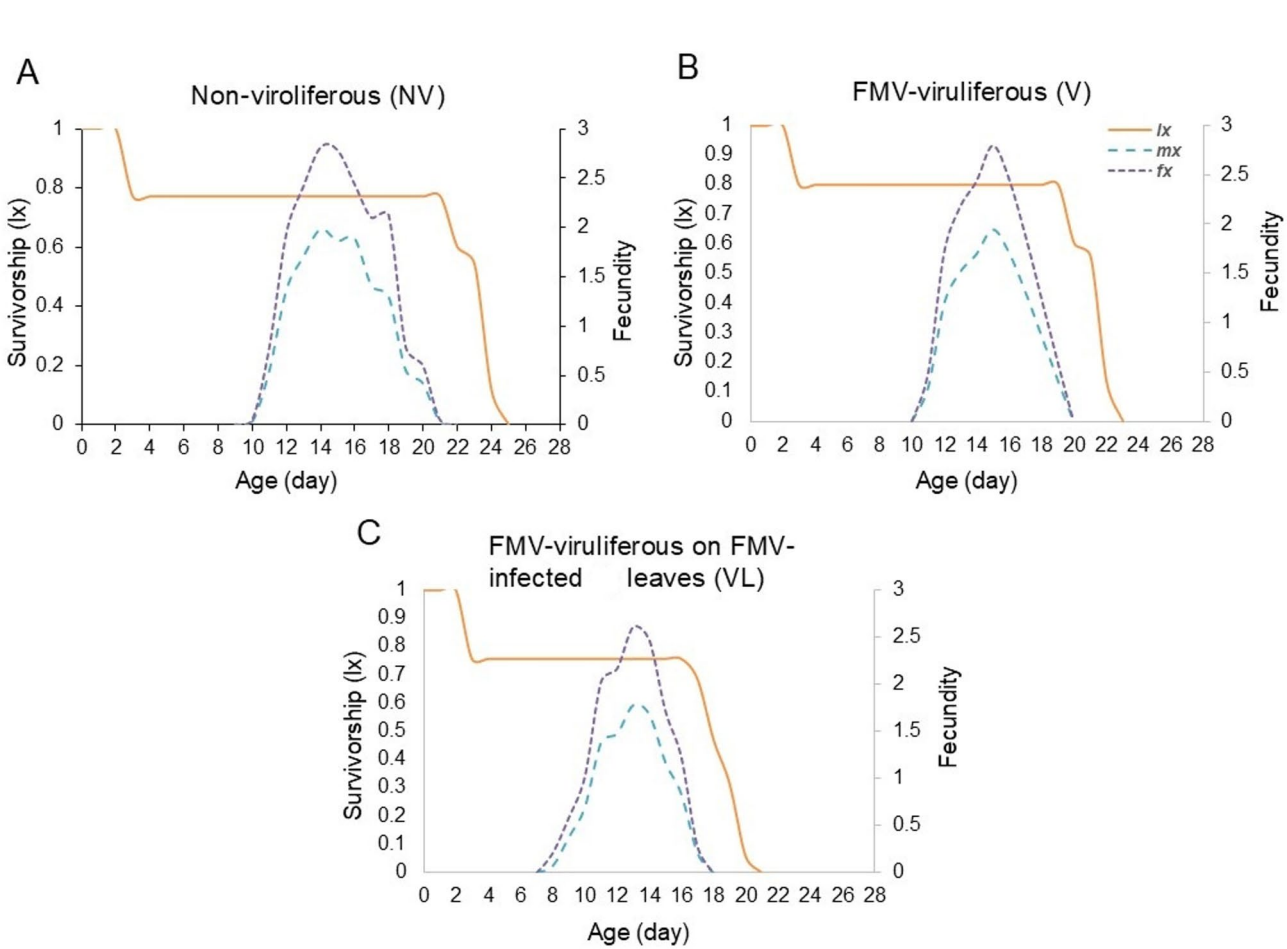
Age-specific survivorship (lx), age-stage fecundity (fx), and age-specific fecundity (mx) of *Aceria ficus*. (**A**) Non-viruliferous (NV), (**B**) viruliferous (V), and (**C**) viruliferous mite reared on FMV-infected fig leaves (VL).

Age-specific survivorship (lx) (Fig. 5A-C) indicates the probability that a newborn will survive. The likelihood of an egg surviving to the adult stage in the non-viruliferous mites was higher than in the viruliferous mites. Also, the non-viruliferous mites survived longer than the viruliferous mites (Fig. 5A-C). Age-specific fecundity (mx) and age-stage-specific fecundity (f_*xj*_) of *A. ficus* are also shown in Fig. 4A-C. As shown, the highest fecundity (18.36 ± 0.09 egg/female) was observed in offspring from non-viruliferous mites and viruliferous mites fed on healthy fig leaves (15.84 ± 0.14 egg/female), while the lowest fecundity (14.19 ± 0.14 egg/female) was observed in the viruliferous mites fed on FMV-infected fig leaves (Fig. 5A-C).

### *Population growth parameters of viruliferous and non-viruliferous A. ficus*

**Fig. 6:**
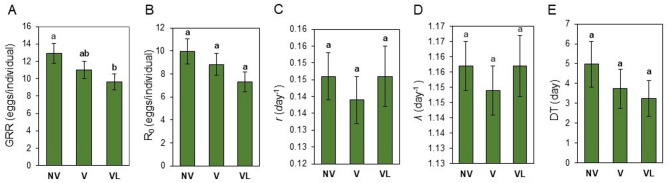
Population parameters (means ± SE) of non-viruliferous (NV), viruliferous reared on healthy fig leaves (V), and viruliferous reared on FMV-infected fig leaves (VL) of *Aceria ficus*, calculated by using the age-stage, two-sex life table. (**A**) Gross reproductive rate (GRR), (**B**) net reproduction rate (R0), (**C**) intrinsic rate of population increase (r), (**D**) finite population growth rate (λ), and (**E**) (T). Different letters indicate significantly different means, using a paired bootstrap test (*P* < 0.05).

Age-stage, two-sex life table parameters were calculated based on data from the entire cohort (Fig. 6A-D). The population growth parameters, including gross reproductive rate (*GRR*), average fertility (*m*_*x*_), and intrinsic rate of increase (*r*) for viruliferous and non-viruliferous populations are shown in Fig. 6A-D. Gross reproduction rate (*GRR*) is the sum of all offspring produced by an individual and was higher in non-viruliferous (12.92 ± 1.14 eggs/individual) populations compared to viruliferous populations reared on infected leaves (9.66 ± 0.91(eggs/individual) (*P* < 0.05) (Fig. 6A), while the viruliferous mites reared on healthy fig leaves showed no differences with the two other populations. The values of the gross reproductive rate (GRR), the net reproductive rate (R_0_), intrinsic (*r*), finite (*λ*) rate of increase, and the average length of a generation (T) were similar between viruliferous and non-viruliferous mites (Fig. 6B-E).

## Discussion

The interaction between plant viruses and their arthropod vectors is a complex and dynamic process that often involves subtle manipulations of the vector’s biology to enhance viral transmission^9^. Such manipulations lead to mutualistic or parasitic interactions depending on the balance between costs and benefits to the vector^5,31^. In this work, we provide information on the interaction between Fig Mosaic Virus and its eriophyid mite vector, *A. ficus*, through a detailed study of the life history traits of both viruliferous and non-viruliferous mites.

Non-viruliferous mites exhibited longer lifespans, greater fecundity, and lower mortality rates compared to viruliferous mites. These findings generally align with the expectation that harboring pathogens imposes physiological costs on the vector, potentially linked to immune responses, resource allocation, or direct viral replication within the mite, which may divert resources from reproduction and survival to managing the infection. Similarly, Southern rice black-streaked dwarf virus (SRBSDV) infection adversely affects the white-backed planthopper (WBPH) by prolonging nymphal stages and reducing survival rates^32^; fecundity is diminished for whiteflies infected with Tomato yellow leaf curl virus^31,33^, and the small brown planthopper, *Laodelphax striatellus* infected with Rice stripe virus^34^. The impact of plant viruses on vectors can vary; for instance, *Bemisia tabaci* feeding on Cucurbit chlorotic yellows virus (CCYV)-infected plants exhibit increased body length, oviposition rates, and extended adult longevity, particularly in females^35^. Similarly, aphid vectors such as *Rhopalosiphum padi* and *Myzus persicae* show improved life history traits when feeding on virus-infected plants^11,36^. The accelerated pre-adult development observed in our study is intriguing and may indicate vector-virus co-evolution. There are evidences that plant viruses can influence vector behavior, physiology, and performance in ways that could indirectly lead to faster development^13,37,38^. This accelerated development could serve as a compensatory mechanism to counteract the reduced survival and fecundity observed in non-viruliferous mites. Quick development might enhance the mite’s ability to reproduce before the onset of virus-induced mortality, thereby maintaining population stability despite the associated fitness costs. Such developmental acceleration aligns with evidence from other plant-virus-vector systems^34,39^, where viral influence adapts vector biology to increase the likelihood of successful transmission^8,9^.

Interestingly, despite clear negative effects on survival and fecundity, the population growth parameters of viruliferous mites did not significantly differ from those of the non-viruliferous mites. This suggests that while individual mites experience fitness losses due to FMV infection, these losses do not translate into substantial changes at the population level. This might denote a sort of population-level buffering effect, with the faster development or altered behavioral traits of the viruliferous mites balancing out, to some degree, the reduced lifespan and fecundity caused by virus acquisition. The absence of significant effects on population growth metrics may also suggest that FMV manipulates *A. ficus* only to the extent needed to facilitate its persistence and transmission without destabilizing the vector population^2^. Therefore, FMV appears to adopt a more neutral strategy, neither significantly enhancing nor severely compromising vector populations. This strategy may reflect the unique biology of eriophyid mites, which are highly specialized and dependent on their plant hosts for survival^21^. The accelerated development of viruliferous mites may partially explain this balance. Faster development could mitigate the negative effects of reduced survival and fecundity by enabling viruliferous mites to reproduce earlier, thereby sustaining population growth. Furthermore, the unique biology of eriophyid mites may restrict how much FMV can influence their fitness without compromising its own transmission efficiency.

This underlines the importance of consideration of the host-plant quality in vector-virus interactions, as FMV-induced changes in fig physiology could further influence mite performance and population dynamics. The striking differences in fitness between viruliferous mites feeding on FMV-infected and healthy fig leaves underscore the role of host plant quality in shaping vector-virus interactions. FMV-infected fig leaves likely underwent physiological changes, making them a less suitable host for *A. ficus*. Such virus-induced changes can further influence vector performance and create indirect fitness costs^40,41^. Virus-induced changes to plant physiology (nutritional composition, defensive chemistry, or tissue structure) are well-established drivers of altered vector performance and behavior and can outweigh direct viral impacts on the vector itself^11,14^. The observed fitness costs associated with FMV infection indicate that viral infection could serve as a selective pressure on *A. ficus*, ultimately driving the evolution of adaptations that alleviate these costs. Over time, this may lead to mite populations developing tolerance or resistance to FMV, which can alter virus transmission dynamics. Similar evolutionary responses have been documented in other vector-virus systems, where vectors evolve traits that minimize the fitness costs of viral infection^9,34^. However, the absence of population-level growth metrics in individual-level fitness costs suggests that *A. ficus* populations may still possess sufficient genetic or phenotypic plasticity to withstand FMV-induced stress without immediate evolutionary adaptation. The identified trade-offs highlight the balanced strategies of FMV, wherein the virus, while imposing individual-level costs on mites, does not destabilize vector populations, thereby preserving its transmission potential. This implies that even reduced mite numbers may sustain FMV transmission and illustrates the complexity of tripartite pathosystems, where often indirect host-mediated effects outweigh direct viral impacts.

## Conclusions

We showed that non-viruliferous mites exhibit shorter immature development, higher offspring production, and higher survival than the viruliferous mites. They also produced a slightly higher number of offspring compared to viruliferous females. These results indicate that while FMV infection imposes fitness costs in *A. ficus* at an individual level, these effects are not scaled up to substantial modifications in population growth rates, suggesting neutrality in the occurrence of FMV in *A. ficus* for the population despite some minor but negligible costs. Understanding this dynamic is critical for devising effective management practices against fig mosaic disease. Further research is essential to understand the underlying molecular mechanisms that influence changes in mite biology and host plant physiology resulting from FMV infection to facilitate the development of targeted interventions.

## Supplementary Information

Below is the link to the electronic supplementary material.

Supplementary Material 1

## Data Availability

Data supporting this study are included within the article.
