# Supplementary material for: Impact of fig mosaic virus on the life history traits and fitness trade-offs of its eriophyid mite vector, Aceria ficus
Source: Sci Rep. 2026 Apr 6;16:16530. doi: 10.1038/s41598-026-46471-4 (PMC13216238; doi:10.1038/s41598-026-46471-4)
Supplement: Supplementary file 1 — Supplementary Material 1 [file 41598_2026_46471_MOESM1_ESM.pdf]

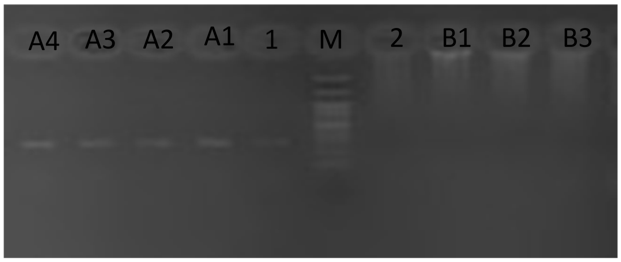

1) Positive Control, 2) negative control, A1 to A4 Viruliferous mite, B1 to B3 Non-viruliferous mite

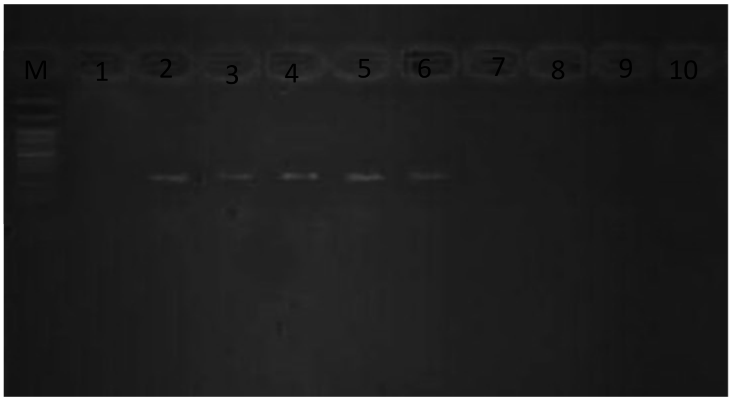

1, Negative control, 2, Positive control, 3 to 6 Infected leaves, 7 to 10 Uninfected leaves
